# The Buffalo Maternity Hospital

**Published:** 1886-05

**Authors:** 


### The Buffalo Maternity Hospital.

The organization of a Maternity Hospital in this city, under the professional management of our skilled obstetricians, and with the liberal spirit which they are sure to infuse into all its details, marks an important addition to our medical institutions. We direct attention to the advertisement in this number of the Journal, which will furnish our readers with the attending and consulting staff. They are among the ablest representatives of the profession of this city. Their social and professional position gives assurance of the success of the institution.

The Maternity Hospital will aim to care for women in childbirth, and also to treat the medical and surgical diseases of women. Ample facilities are furnished for parturient women, as well as for gynæcological cases.

The Hospital commends itself to the profession. It aims to be another of the many agencies in this city for the alleviation of physical suffering and the extension of human life. We congratulate our editorial confrere, Dr. Lothrop, the physician-in-chief, upon the enterprise manifested in this excellent work.

